# Knowledge mapping of autistic traits: a visual analysis via CiteSpace

**DOI:** 10.3389/fpsyt.2025.1522299

**Published:** 2025-04-24

**Authors:** Fu-Qiang Qiao, Tong-Tong Du, Yingao Guo, Ya-Jie Dong, Si-Ning Li, Xu Qiang, Duan-Wei Wang

**Affiliations:** ^1^ School of Education and Psychology, University of Jinan, Jinan, China; ^2^ Tianjin Yang Jialou Senior High School, Tianjin, China; ^3^ Department of Psychology, Ningbo University, Ningbo, Zhejiang, China; ^4^ Shandong Mental Health Center, Shandong University, Jinan, Shandong, China

**Keywords:** autistic traits, Citespace, visual analysis, autism spectrum disorder, (WOS) web of Science

## Abstract

**Objective:**

The aim of this study was to analyze the research hotspots and frontiers in the field of autistic traits (AT) between 1997 and 2024.

**Methods:**

The Web of Science Core Collection (WOSCC) is used as a data source in the study, analyzing 1,044 academic articles on AT published between 1997 and 2024. The included articles were visually analyzed using CiteSpace 6.2.R4 software, which is used to map keywords and core literature related to AT and to analyze research progress and emerging hotspots in the field

**Results:**

A total of 1,044 articles have been included in the analysis, and the total number of articles has demonstrated an upward trajectory. The nations of England, the USA, and Australia are at the vanguard of this body of literature. With regard to research institutions, the University of London has attracted considerable attention as a result of its substantial contributions to the field of AT. The most relevant research is published in the Journal of Autism and Developmental Disorders.

**Conclusion:**

The scope of AT research has expanded considerably, encompassing psychology, education, and other disciplines. The research dimensions extend beyond behavioral, genetic, cognitive, and neural aspects to include environmental and hormonal factors. Furthermore, the study population has evolved from twins and the general population to focus on specific groups, such as individuals with psychiatric disorders. This broadening of focus has led to a significant increase in AT research in recent years. In sum, this study provides a comprehensive perspective that can inform and guide further in-depth research on AT.

## 1 Introduction

Autistic traits (AT) are characteristics that resemble, though are less pronounced than, the behavioral manifestations and personality traits observed in individuals with autism. These traits encompass a range of social interactions and social-emotional competencies, including indifference, unsophistication, subtlety, unresponsiveness, and a lack of emotional response and empathy. Autism spectrum disorder (ASD) is a neurodevelopmental condition characterized by persistent deficits in social communication and interaction across multiple contexts, along with repetitive and stereotyped behaviors, narrow interests, and restricted activities (1). Autistic traits are increasingly described as a collection of behavioral, personality, and cognitive characteristics associated with ASD (2). These traits are found in both individuals with ASD and the general population, though they differ quantitatively in severity (3). Those with high levels of AT and individuals with ASD share similarities in various emotional, cognitive, and behavioral domains, including elevated anxiety, impaired cognitive flexibility, and deficits in social skills (4–7).

The earliest research on AT dates back to a 1997 study, which provided a detailed characterization of learning disabilities, revealing that adults with such disabilities frequently exhibit these traits. Furthermore, the study indicated that younger individuals and those with severe or pervasive learning disabilities are more likely to manifest AT (8). Early research efforts exhibited a relatively narrow focus, primarily concentrating on twin samples, the general population, and individuals diagnosed with ASD (9, 10). Early research also focused on measuring AT, with a greater emphasis on the assessment tools themselves. Specifically, studies explored the use of instruments such as the Social Responsiveness Scale (SRS) and the Autism-Spectrum Quotient (AQ), including various iterations of the AQ, during the period of 2003-2006 (11–13).

In 2008, a seminal publication elucidated the “fractionable autism triad” through behavioral, genetic, cognitive, and neural evidence. This work provided a framework, encompassing these four dimensions. (14). Regarding behavioral dimensions, for instance, research might explore how AT predicts behavioral outcomes (15). In the realm of genetics, studies could examine associated genes (16). Furthermore, these dimensions can be integrated in research. For instance, studies have investigated the influence of genetics on behavior within the genetic and behavioral dimensions (17). Similarly, the cognitive and behavioral dimensions can be studied in conjunction (18).However, the research extends beyond these four dimensions, encompassing additional dimensions such as environmental and emotional factors. The extant literature on AT is expanding, with research increasingly focusing on specific populations. For example, research on cohorts with obsessive-compulsive disorder, anorexia nervosa, attention-deficit/hyperactivity disorder, bipolar affective disorder, and post-traumatic stress disorder commonly examines behavioral, genetic, cognitive, and neurological dimensions (19, 20).

Literature reviews, a vital component of educational and scientific research, play a crucial role in summarizing and synthesizing research findings (21, 22). Currently, a lack of bibliometric analysis on AT leave uncertainties regarding the focus of current research and the overall evolution in the field. This study utilizes CiteSpace to graphically examine various aspects of research on AT, including the annual number of publications, authors, institutions, countries, journals, cited references, and keywords. This study not only provides valuable guidance for future research and application but also offers a much-needed methodological framework for understanding the development of the field.

## 2 Methods

### 2.1 Data source

In the current study, several databases were adopted. Web of Science (WoS) is one of the most well-known scientific citation index databases in the world. Publications included in the Web of Science Core Collection (WoSCC) are considered an essential part of the research process. In 2020, the Web of Science Core Collection will contain more than 74.8 million scientific records and 1.5 billion references in 254 subject areas dating back to 1900 (23–25). Therefore, in this study, we chose the WoSCC database as our primary data source.

### 2.2 Search strategy (**Figure 1**)

**Figure 1:**
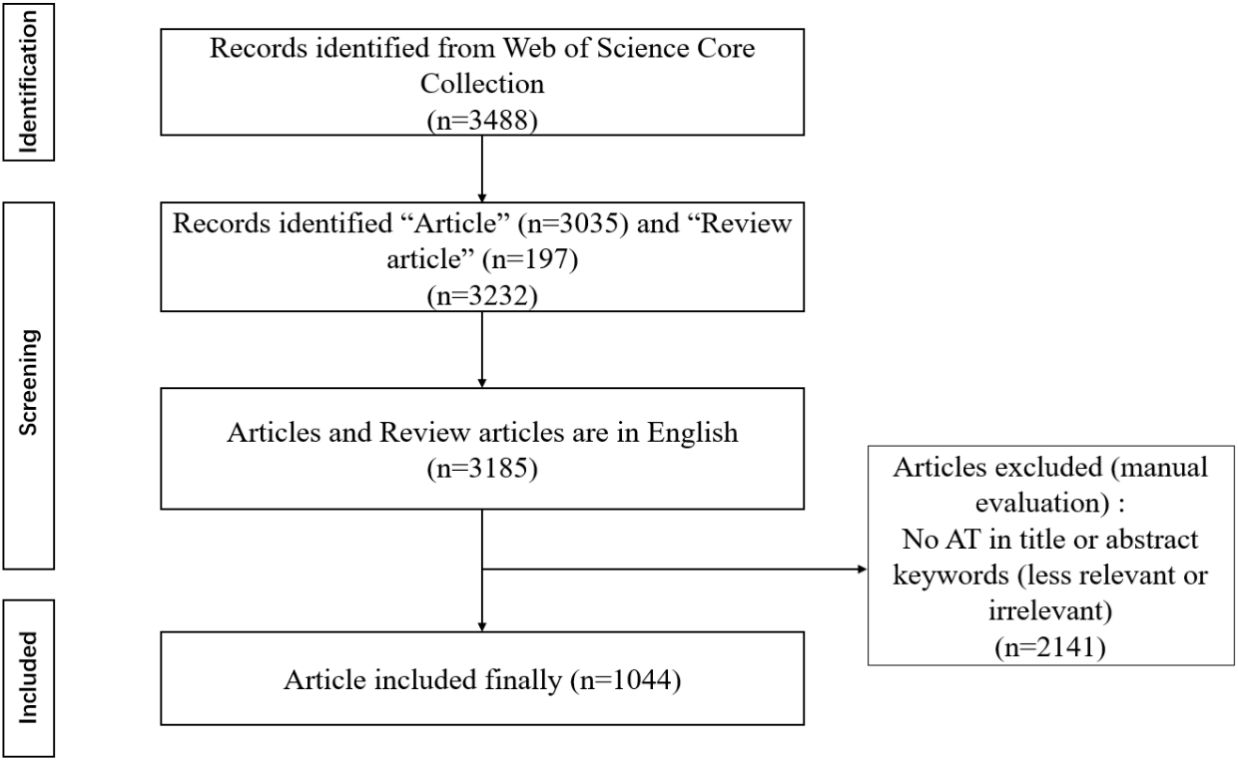
Flow diagram for screening articles.

All data were retrieved from WoSCC on July 25, 2024, using a four-component approach: (i) topic = autistic traits; (ii) document type = “article” or “review article”; (iii) language = English. The plain text format of complete records and their corresponding cited references were downloaded for further examination.

#### 2.2.1 Inclusion criteria

Inclusion criteria were: (i) articles extracted from the WoSCC; (ii) articles or review articles; (iii) articles written in English; (iv) articles on AT.

#### 2.2.2 Exclusion criteria

Exclusion criteria were as follows: (i) hand-collected articles; (ii) articles not formally published; (iii) conference abstracts and proceedings; (iv) duplicate publications or the same study; (v) articles unrelated to the topic; and (vi) articles not written in English.

### 2.3 Analysis tool

CiteSpace is a valuable and essential tool for analyzing scientific literature. It is primarily used to evaluate the corpus of papers within a specific field and to illustrate the structure and trajectory of research in that domain, facilitating the identification of patterns, hotspots, and the evolution of knowledge over time (26, 27). In this study, CiteSpace V.6.2.R4 (64-bit) was applied to analyze research related to AT. The goal was to provide evidence-based support for researchers, gain insights into the current state and trends within the field, and generate new ideas for future development.

### 2.4 Data analysis

CiteSpace is specifically designed to facilitate the detection of emerging trends and abrupt changes in the scientific literature (28). In this study, the CiteSpace software was used to identify citation bursts across multiple dimensions, such as research publication year, author, research institution, journal, country, keywords, and hotspots.

## 3 Results

### 3.1 Annual publication analysis

**Figure 2:**
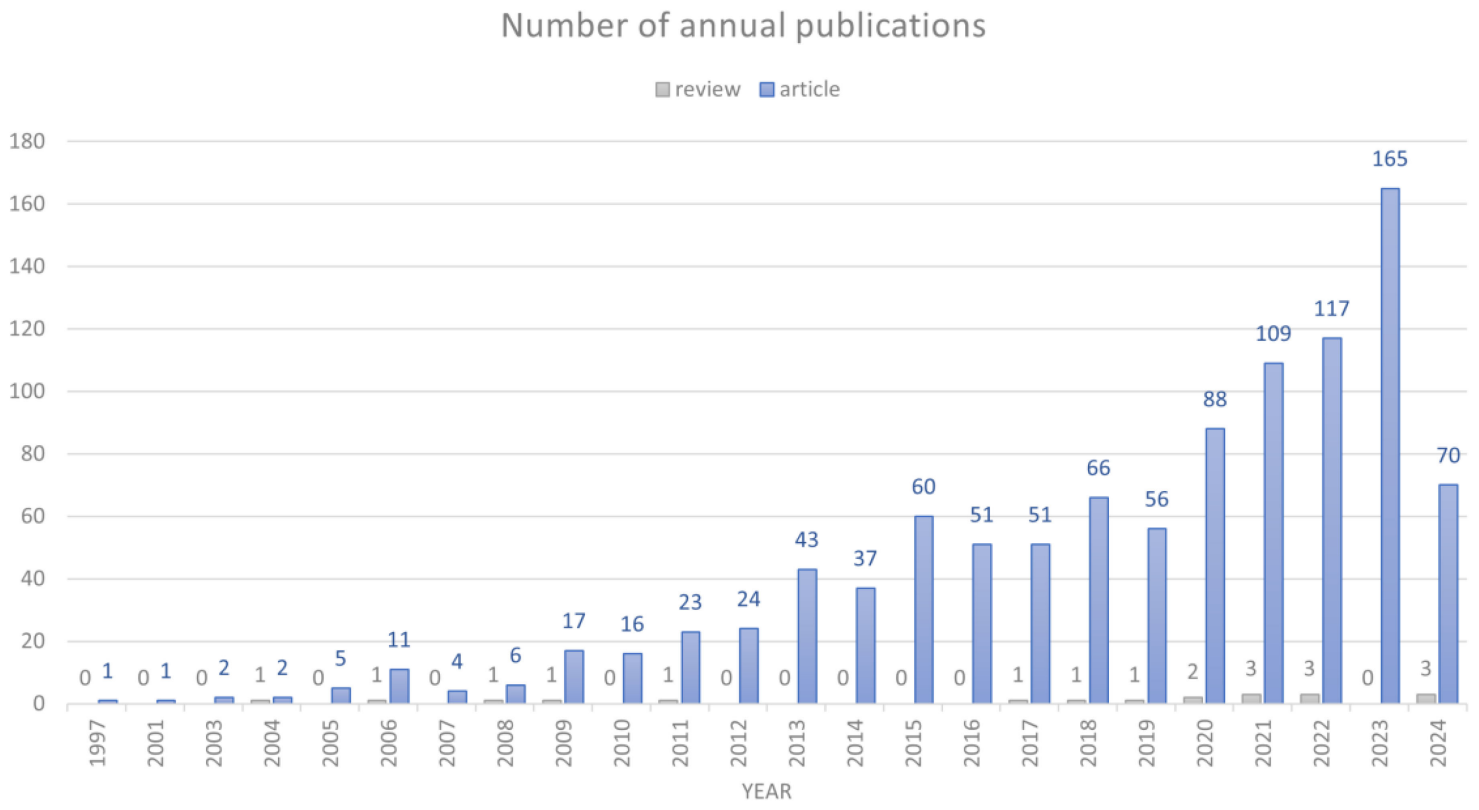
The number of annual publications and growth trends of Autistic Traits (AT) from 1997 to 2024.

Using the specified search strategy, a total of 1,044 publications meeting the inclusion criteria were retrieved. **Figure 2** illustrates the annual number of articles published on AT. The document types are divided into two categories: articles and reviews. As can be seen, the number of reviews is relatively small. The first qualifying article on AT was published in 1997. Between 1997 and 2012, the number of articles on AT remained relatively low, with fewer than 30 articles published per year. Starting in 2012, there was a gradual increase in the number of AT articles, with notable surges observed between 2019 and 2020, and again between 2022 and 2023. The total number of articles published from 1997 to 2019 is less than the number of articles published in the most recent five-year period. It should be noted that the count of AT articles for 2024 is limited due to data collection being conducted in July of that year. This trend indicates that research on AT is currently a prominent focus, with the majority of articles published in the past five years.

### 3.2 Cited journals analysis

**Figure 3:**
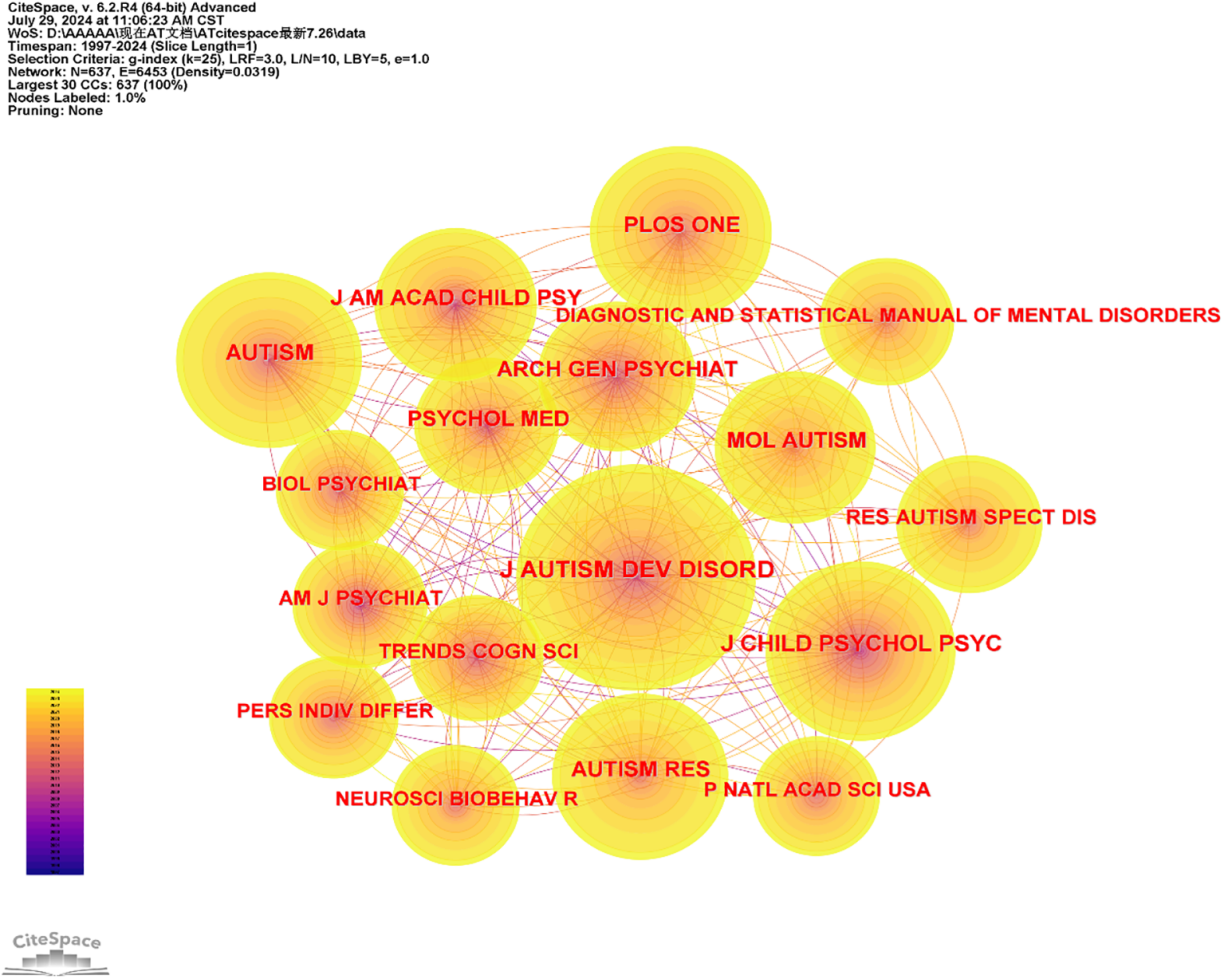
Co-occurrence map of cited journal of AT from 1997 to 2024.

**Figure 4:**
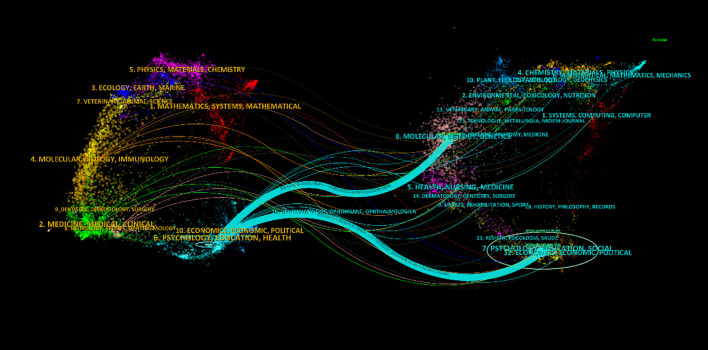
A dual-map overlays of the journals of AT research.

Analyzing the journals cited in the field of AT provides valuable insights into key sources of knowledge dissemination and facilitates the efficient location of relevant information. In this study, articles on AT published between 1997 and 2024 were analyzed at one-year intervals. The cited journals were categorized as nodes, resulting in a distribution map with a merged network comprising 637 nodes and 6,453 links, as illustrated in **Figure 3**. In this network, journals are represented as nodes and their interconnections are depicted as lines. Nodes with higher frequency are considered more influential and impactful in the development of the scientific field. The thickness of the lines between nodes reflects the strength of their relationships. **Figure 4** presents a dual-map overlay of journals, showing the relationships between cited journals on the left and citing journals on the right. The majority of journals are focused on the field of psychology, while others cover topics such as education, health, and social issues. **Table 1** lists the top ten most cited journals in this field. The *Journal of Autism and Developmental Disorders* ranks first with 976 publications, followed by the *Journal of Child Psychology and Psychiatry* with 604 publications, and *Autism* with 592 publications. *PLOS ONE* and *Autism Research* are the fourth and fifth most cited journals, respectively. It is noteworthy that most of the top ten journals by number of articles are from the United States.

**Table 1:** The top ten cited journal and centrality in the research field of Autistic Traits (AT) from 1997 to 2024.

Ranking	Cited Journal	Frequency	Centrality	Half-life
1	J AUTISM DEV DISORD	976	0.02	22.5
2	J CHILD PSYCHOL PSYC	604	0.01	17.5
3	AUTISM	592	0.02	18.5
4	PLOS ONE	524	0.01	10.5
5	AUTISM RES	514	0.01	10.5
6	ARCH GEN PSYCHIAT	451	0.01	17.5
7	J AM ACAD CHILD PSY	450	0.01	18.5
8	MOL AUTISM	417	0.02	8.5
9	PSYCHOL MED	366	0.01	18.5
10	RES AUTISM SPECT DIS	361	0.01	9.5

### 3.3 Countries analysis

**Figure 5:**
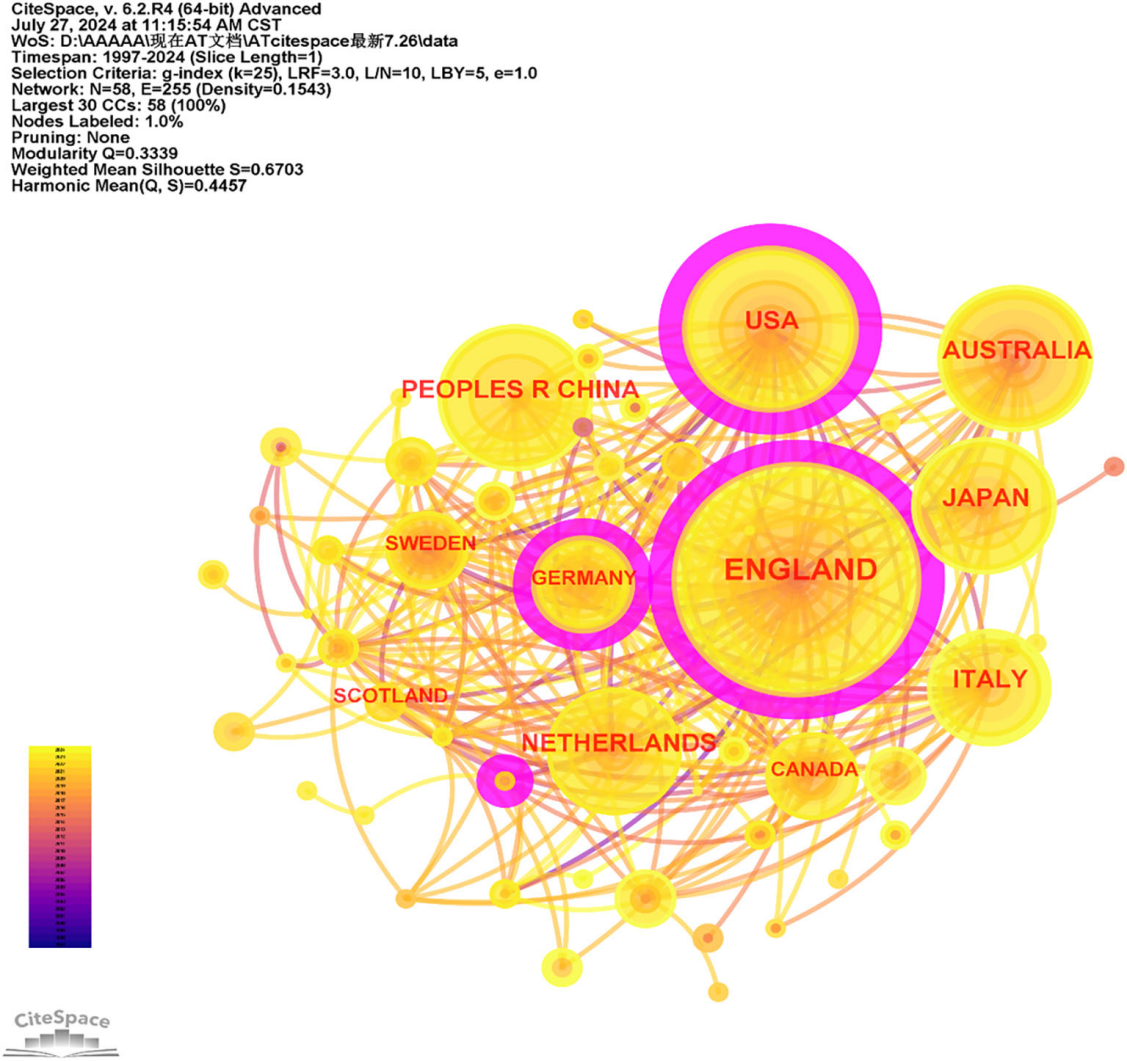
Co-occurrence map of countries of AT from 1997 to 2024.

To examine the connections between articles published in different countries, we analyzed all articles on AT from 1997 to 2024 at one-year intervals. This analysis produced a country-specific distribution map, shown in **Figure 5**, which displays a merged network consisting of 58 nodes and 255 links. In this network, nodes represent countries, and the lines illustrate the relationships between them. The size of the nodes reflects the number of publications, with larger nodes indicating higher publication productivity.

**Table 2** lists the top ten most productive countries in AT research. England leads with the highest publication productivity, totaling 302 articles, followed by the USA with 197 articles. These are the only two countries with more than 150 articles. Australia, Japan, China, and the Netherlands follow.

**Table 2:** The top ten countries and centrality in the research field of AT from 1997 to 2024.

Ranking	Countries	Frequency	Centrality	Half-life
1	ENGLAND	302	0.35	22.5
2	USA	197	0.33	14.5
3	AUSTRALIA	124	0.05	9.5
4	JAPAN	115	0.08	14.5
5	PEOPLES R CHINA	104	0	9.5
6	NETHERLANDS	92	0.05	13.5
7	ITALY	92	0.08	10.5
8	GERMANY	57	0.11	12.5
9	CANADA	55	0.08	15.5
10	SWEDEN	53	0.09	15.5

### 3.4 Institutional analysis

**Figure 6:**
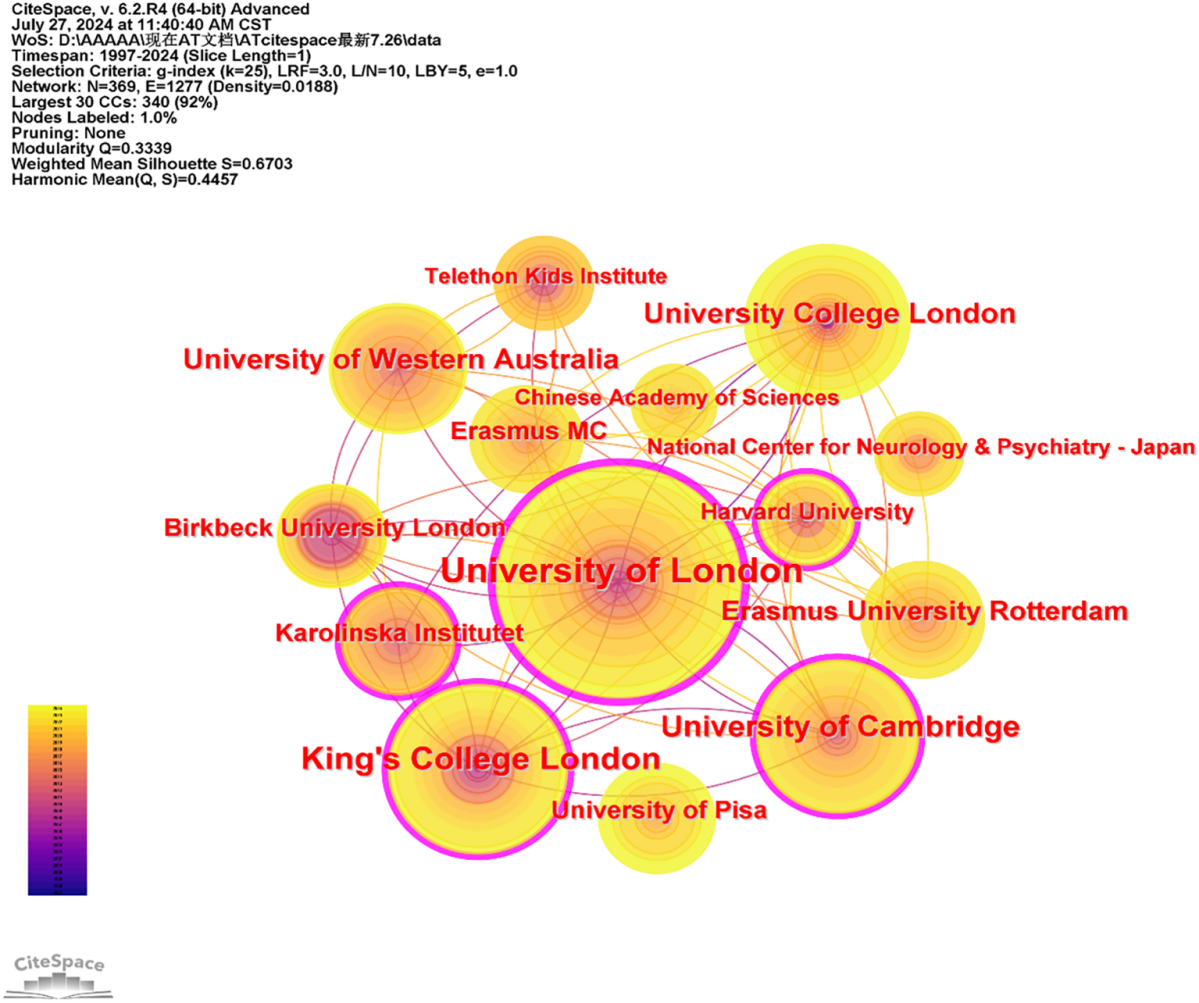
Co-occurrence map of institutions of AT from 1997 to 2024.

CiteSpace was employed to generate a network map to explore collaborative relationships between institutions and to identify influential institutions in the field of AT. The node type was set to institution, resulting in a distribution map comprising 369 nodes and 1,277 links, as shown in **Figure 6**. In this map, nodes represent institutions, and lines depict the relationships between them. Institutions with a higher number of published papers are generally considered more important and influential.

**Table 3** presents the top ten most productive institutions in this field. The University of London ranks first with 129 publications, followed by King’s College London with 73, and the University of Cambridge in third place with 69. Most of the top ten institutions are located in England, highlighting the country’s prominent role in the field. Additionally, the close connections between major institutions indicate strong collaborative networks.

**Table 3:** The top ten institutions and centrality in the research field of AT from 1997 to 2024.

Ranking	Institutions	Frequency	Centrality	Half-life
1	University of London	129	0.38	14.5
2	King’s College London	73	0.14	13.5
3	University of Cambridge	69	0.18	13.5
4	University of Western Australia	54	0.05	7.5
5	University College London	48	0.06	15.5
6	Erasmus University Rotterdam	34	0.02	6.5
7	Karolinska Institute	33	0.11	6.5
8	Erasmus MC	32	0.02	6.5
9	Birkbeck University London	30	0.04	3.5
10	University of Pisa	27	0.01	3.5

### 3.5 Authors analysis

**Figure 7:**
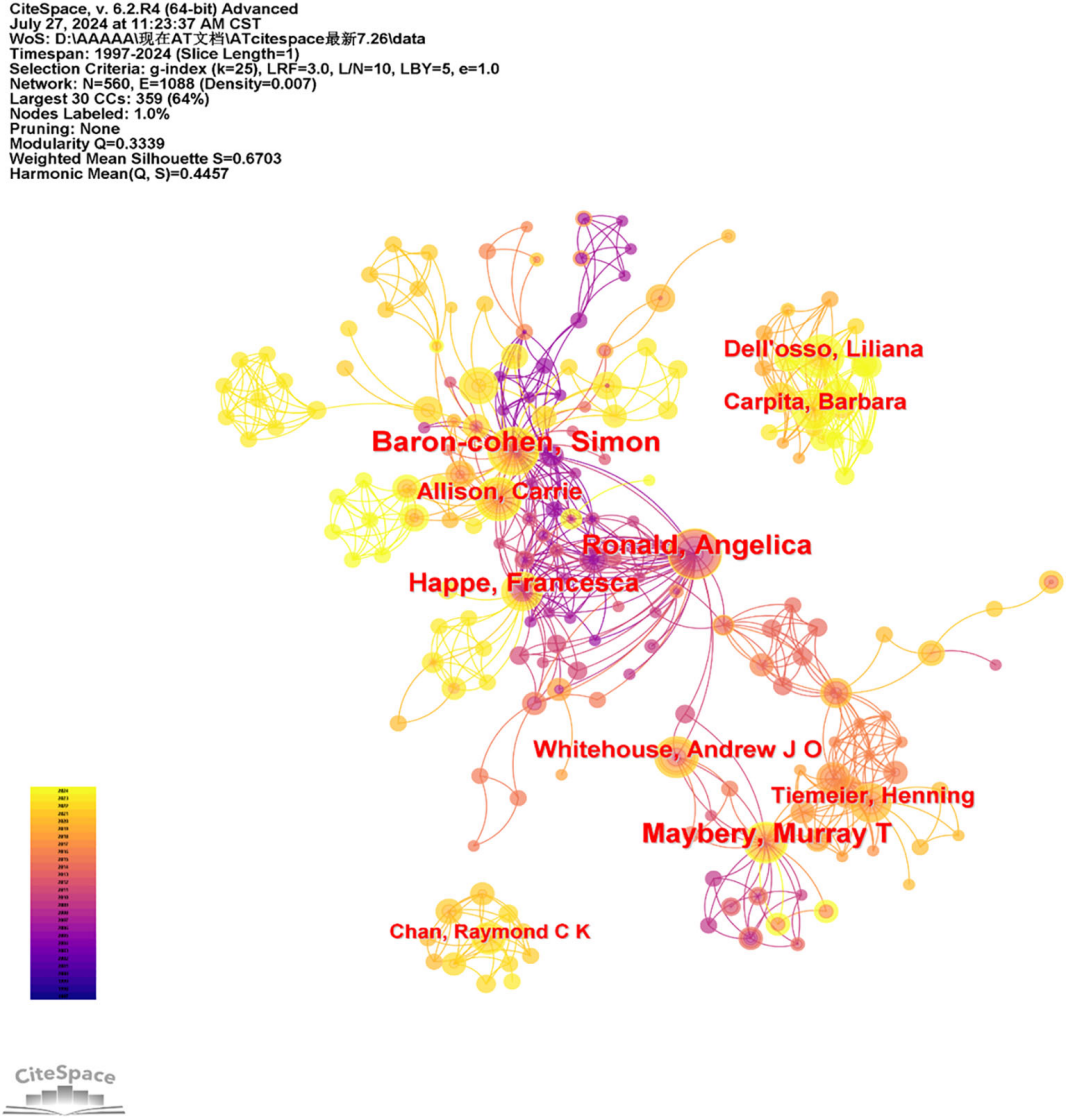
Co-occurrence map of author of AT from 1997 to 2024.

The articles selected for analysis spanned from 1997 to 2024. Authors were designated as the node type to create a network map of co-authorship, as illustrated in **Figure 7**. This merged network map comprises 560 nodes and 1,088 links. In the map, nodes represent authors, and lines indicate their collaborative relationships. The size of each node corresponds to the author’s publication output, with larger nodes reflecting a higher number of published articles.

Among the active authors, Simon Baron-Cohen from the Autism Research Centre at the University of Cambridge ranks first with 38 publications. Other notable authors include Ronald Angelica from the University of London, Murray T. Maybery from the University of Western Australia, Francesca Happe from King’s College London, and Carrie Allison from the University of Cambridge. **Table 4** lists the top ten most productive authors in this field. Many of these authors are from the United Kingdom and are trained psychologists, reflecting a strong network of collaboration within this region.

**Table 4:** The top ten author and centrality in the research field of AT from 1997 to 2024.

Ranking	Author	Frequency	Centrality	Half-Life
1	Baron-cohen, Simon	38	0.08	10.5
2	Ronald, Angelica	33	0.07	4.5
3	Maybery, Murray T	30	0.03	7.5
4	Happe, Francesca	28	0.02	5.5
5	Allison, Carrie	19	0.01	10.5
6	Whitehouse, Andrew J O	18	0.02	6.5
7	Dell’osso, Liliana	17	0	3.5
8	Carpita, Barbara	16	0	3.5
9	Tiemeier, Henning	16	0.01	2.5
10	Chan, Raymond C K	12	0	1.5

### 3.6 Cited references analysis

**Figure 8:**
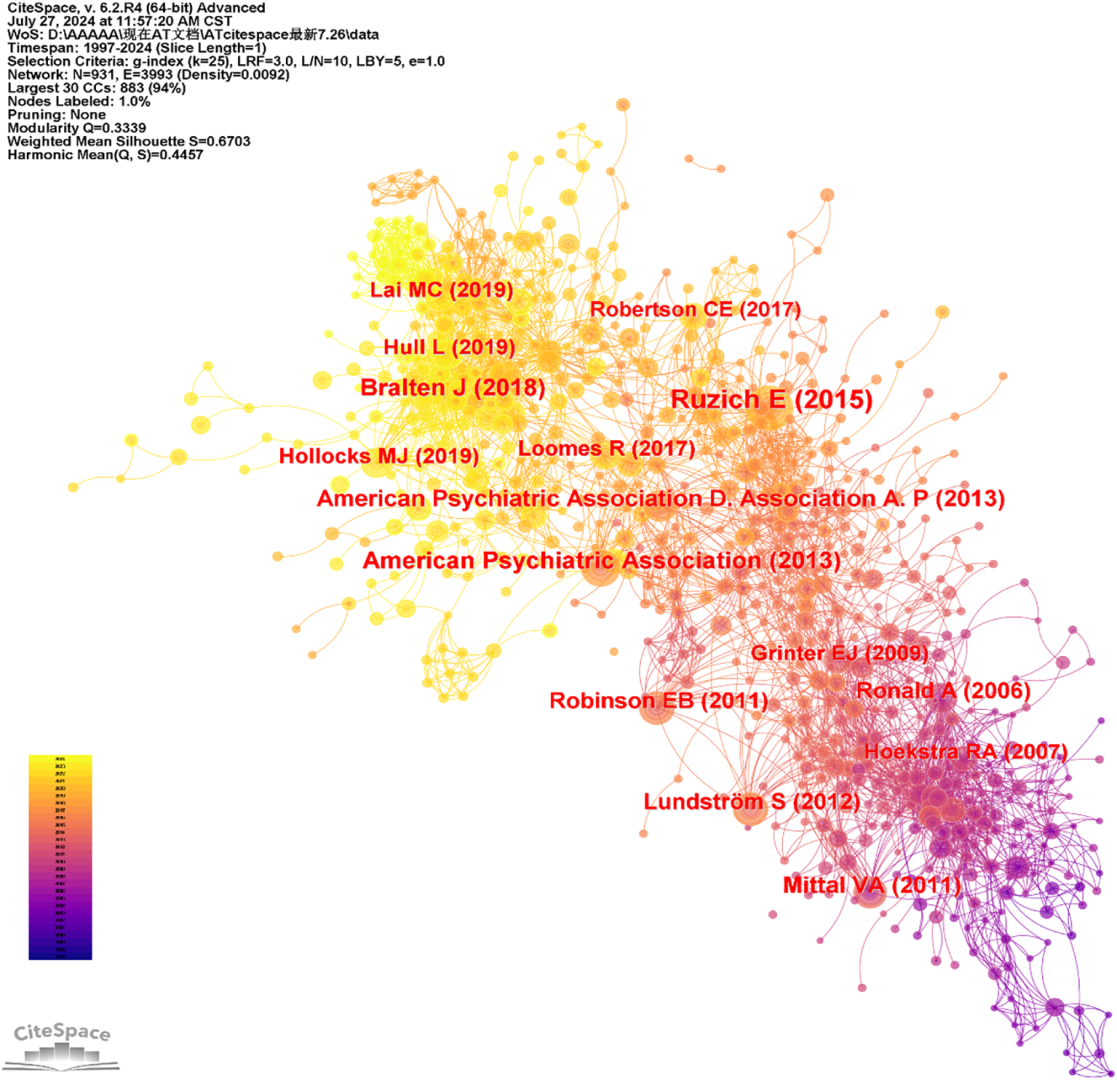
Co-occurrence map of cited references of AT from 1997 to 2024.

Co-citation analysis is a method used to measure the relationships between articles based on their simultaneous citation by other articles, indicating a co-citation relationship (29). For analysis, references were designated as the node type in CiteSpace, with articles published between 1997 and 2024. This approach generated a co-citation network map, revealing a merged network of 931 nodes and 3,993 links, as shown in **Figure 8**. In this network, nodes represent co-cited references, and lines describe the relationships between these co-citations.

**Table 5** presents the top ten most cited references in this study. The most cited literature is “Measuring Autistic Traits in the General Population: A Systematic Review of the Autism-Spectrum Quotient (AQ) in a Nonclinical Population Sample of 6,900 Typical Adult Males and Females” by Ruzich et al. (30). This is followed by “Autism Spectrum Disorders and Autistic Traits: Shared Genetics and Biology” by Bralten et al. (31) and the “Diagnostic and Statistical Manual of Mental Disorders” by the American Psychiatric Association (1). The majority of co-citations focus on the academic performance and diagnosis of AT.

**Table 5:** The top ten cited reference and centrality in the research field of AT from 1997 to 2024.

Ranking	Cited references	Year	Frequency
1	Measuring autistic traits in the general population: a systematic review of the Autism-Spectrum Quotient (AQ) in a nonclinical population sample of 6,900 typical adult males and females	2015	51
2	Autism spectrum disorders and autistic traits share genetics and biology	2018	37
3	American Psychiatric Association (1)	2013	35
4	Diagnostic and Statistical Manual of Mental Disorders	2011	30
5	American Psychiatric Association D. Association A. P (1)	2013	29
6	Evidence that autistic traits show the same etiology in the general population and at the quantitative extremes (5%, 2.5%, and 1%).	2011	28
7	Autism Spectrum Disorders and Autistic like Traits.	2012	28
8	Genetic Heterogeneity Between the Three Components of the Autism Spectrum: A Twin Study.	2006	27
9	Development and Validation of the Camouflaging Autistic Traits Questionnaire (CAT-Q).	2019	24
10	Heritability of Autistic Traits in the General Population.	2007	23

### 3.7 Keywords analysis

**Figure 9:**
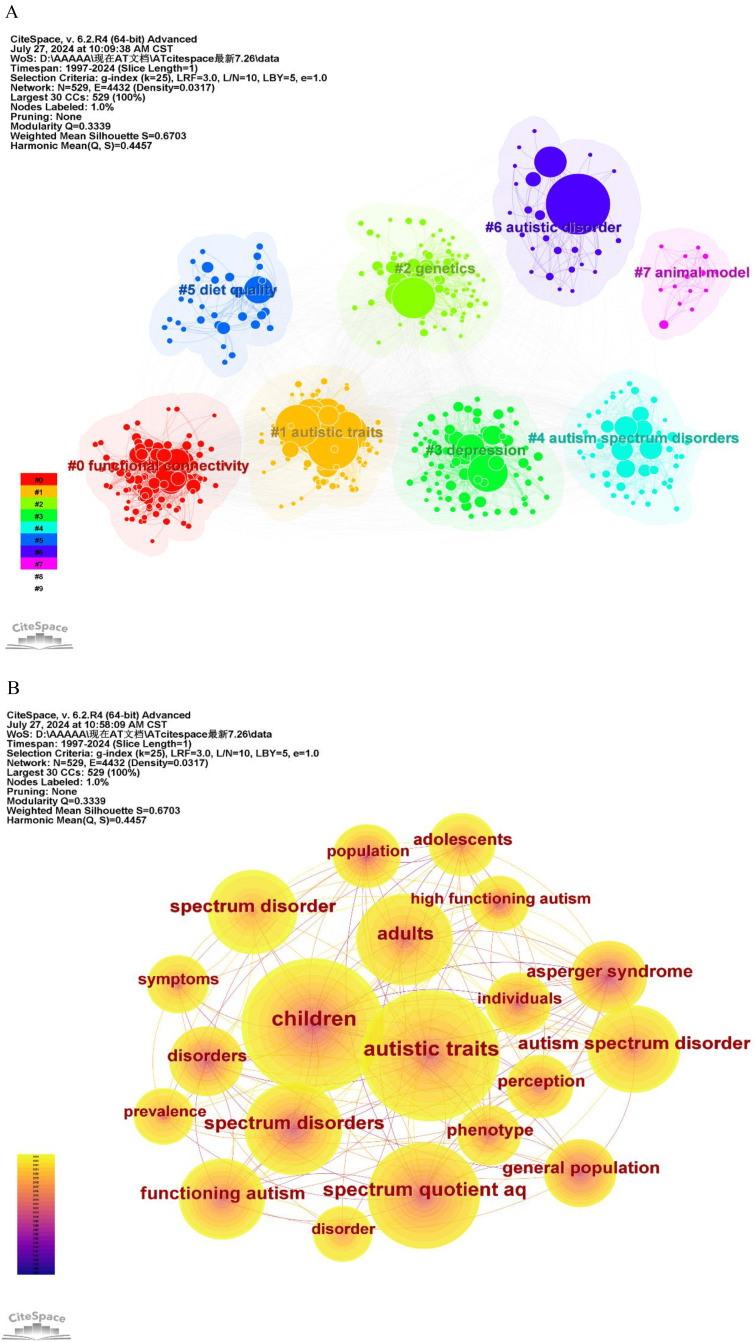
**(A)** The clustering map of keywords of AT from 1997 to 2024; and **(B)** The co-occurrence map of keywords of AT from 1997 to 2024.

**Figure 10:**
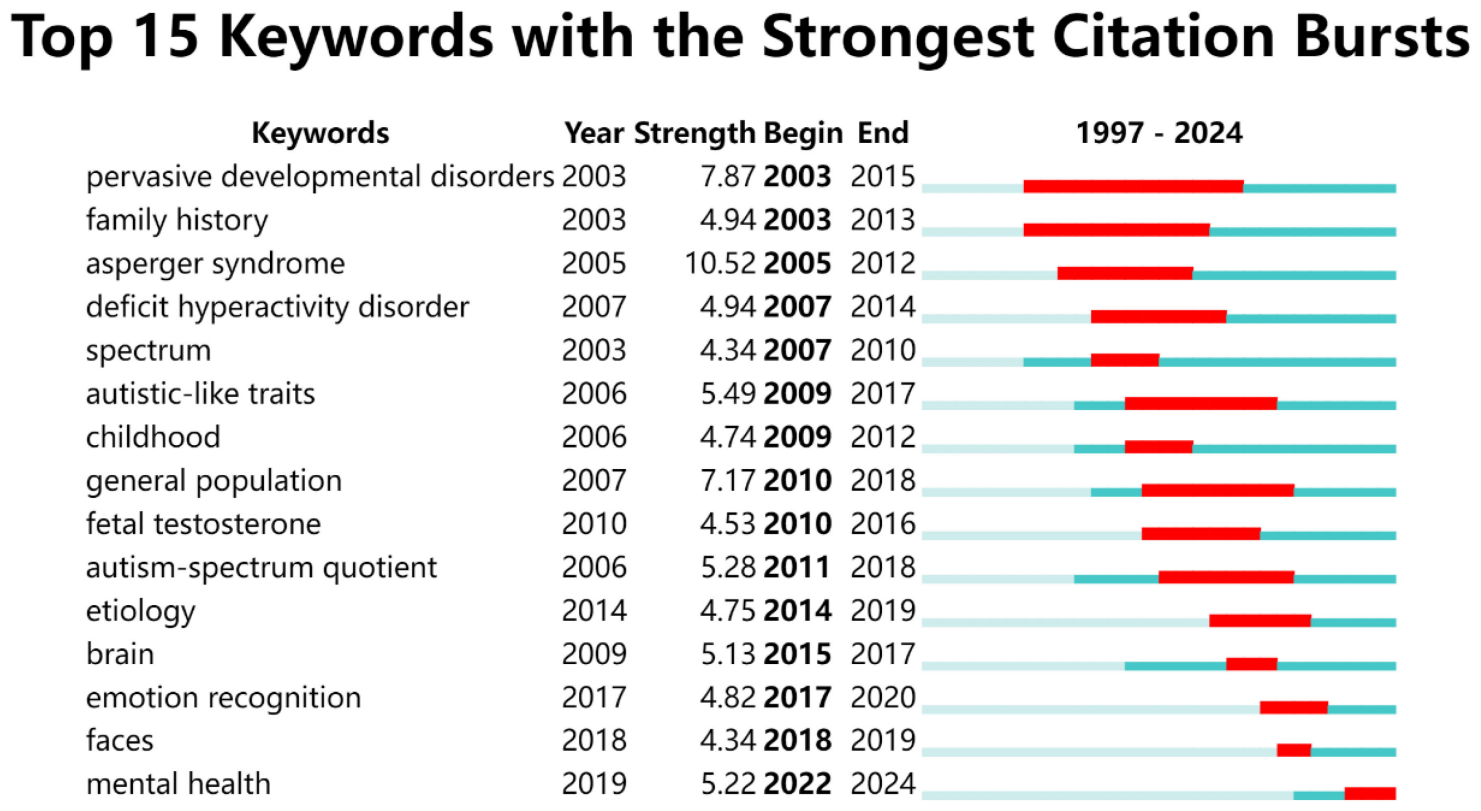
Visualization of top 15 keywords with the strongest citation bursts of AT from 1997 to 2024.

Keywords are essential for understanding the core topics of a study. Analyzing keywords allows us to summarize research themes, identify hotspots, and explore research directions (32). For this analysis, articles published between 1997 and 2024 were selected with a one-year time slice, and keywords were used as the node type in CiteSpace. This generated a keyword co-occurrence map comprising 529 nodes and 4,432 links, as shown in **Figures 9A, B**. The most frequently occurring keywords in this study include “children” (389 occurrences), “autistic traits” (366), “spectrum quotient AQ” (240), “adults” (187), “spectrum disorders” (181), “autism spectrum disorder” (161), “spectrum disorder” (150), “functioning autism” (144), and “Asperger syndrome” (127). A timeline based on keyword interactions and relationships within the field is constructed to explore the evolutionary trajectory and characteristics of the research domain. **Figure 10** displays the top 15 keywords with the strongest citation bursts. The blue line represents the time intervals, while the red line indicates the periods of significant keyword bursts. In AT research, earlier keywords include “pervasive developmental disorders”, “family history,” and “asperger syndrome”. More recent keywords include “autism-spectrum quotient”, “mental health”, “brain” and “emotion recognition”. Additionally, terms such as “autistic-like traits” and “general population” show significant impact.

## 4 Discussion

### 4.1 Trends on AT

The analysis encompasses various aspects of the study of AT, including publications, journals, countries, institutions, authors, co-cited references, and keywords, as represented in CiteSpace. Notably, the seminal article on AT, “Autistic Traits in Adults with Learning Disabilities,” published in 1997, appears to have marked the beginning of this research area. This study explored the prevalence of AT and their association with maladaptive behaviors among adults with learning disabilities in a specific geographical region. It found that AT were prevalent in this population, and individuals with a higher number of these traits were more likely to be severely learning disabled and exhibit a wide range of challenging behaviors.

The growth trend in AT publications from 1997 to 2024 can be divided into two distinct phases. The first phase (1997 to 2012) is characterized by stagnation or slow emergence. The peak number of publications in this phase was 24, recorded in 2011 and 2012. The total number of publications during this phase was 117, indicating relatively modest growth. The second phase (2013 to 2024) represents a period of significant growth and rapid development. The data collection for 2024, which was conducted in July, indicates a preliminary count of 73 publications for that year. The total number of publications from 2013 to 2024 is 927, approximately nine times the number from the first phase. In particular, the number of articles published each year after 2020 shows a significant increase compared to the previous phase, with exceptional numbers in 2022 (120) and 2023 (165).

The presence of AT has been demonstrated not only in individuals diagnosed with ASD but also in the general population, where AT can be observed at subthreshold levels (33). Additionally, subsequent research has shown that other populations, including psychiatric patients with various disorders and participants in scientific courses, are also more likely to exhibit AT (3, 34–40). The combined presence of elevated schizotypal and autistic traits was associated with an even greater reduction in social functioning (41). Future research in the field of AT should continue to prioritize the study of specific populations, such as individuals with psychiatric disorders and those involved in criminal activities. This broadening of focus has led to a significant increase in AT research in recent years.

### 4.2 Research focus and hotspots on AT

#### 4.2.1 Measuring tools for AT

The Social Responsiveness Scale (SRS) and the AQ have been widely recognized as valid instruments for assessing clinically significant AT. The SRS, formerly known as the Social Reciprocity Scale, gathers “first-hand” ratings from individuals (parents or teachers) who have observed the child in naturalistic social settings (12). The AQ was developed to measure the extent to which an adult of normal intelligence exhibits autistic traits (13). AQ has been widely adopted in the literature, with the term “spectrum quotient aq” appearing 240 times, ranking third in keyword frequency (see **Figure 9B**). A quantitative analysis of citations reveals a focus related to AT. The most cited study in this field is “Measuring Autistic Traits in the General Population: A Systematic Review of the Autism-Spectrum Quotient (AQ) in a Nonclinical Population Sample of 6,900 Typical Adult Males and Females” by Ruzich et al. (30), which has garnered 51 citations. This study underscores the significance of the Autism-Spectrum Quotient (AQ) in assessing AT.

The AQ has been administered to adults with autism who are of at least average intelligence, as well as to non-clinical controls and clinical control groups, including individuals with schizophrenia, prosopagnosia, anorexia, and depression. It is widely referenced across various disciplines, remaining a valuable tool for assessing AT in individuals with typical cognitive abilities (30). Researchers often assess the validity of these tools when conducting cross-cultural studies (42). Furthermore, alternative assessment instruments, such as the SCDC (the Social and Communication Disorders Checklist), are also employed (43), and the development of more effective measurement tools is encouraged.

#### 4.2.2 Subthreshold autistic traits

AT are continuously distributed and exhibit moderate to high heritability (9, 12, 44), and AT are present in both individuals diagnosed with ASD and the general population, albeit at subthreshold levels (33). Jian and Xudong (45) noted that AT can be classified as suprathreshold or subthreshold (autistic-like traits), depending on whether symptom severity meets clinical diagnostic criteria. The initial investigation of first-degree relatives of individuals with ASD aimed to identify subthreshold autistic traits, also referred to as the “broad autism phenotype” (BAP) (46, 47). Many typical relatives of individuals with ASD exhibit behaviors similar to those with ASD but do not meet the criteria for a clinical diagnosis. These mild social and communicative impairments, along with personality and cognitive traits resembling those seen in ASD, are collectively referred to as the broad autism phenotype (48–50).

The continuum of subthreshold autistic traits includes both the BAP and traits found within the broader non-clinical population. However, the BAP, particularly in relatives of individuals with ASD, is commonly used in genetic studies and is associated with higher levels of subthreshold traits than the general population (51). Researchers used the Autism Spectrum Quotient (AQ) to assess the prevalence of BAP, aiming to determine whether parents of autistic children differ from parents of typically developing children (TDC) in their AQ scores. The findings support the hypothesis that social and communication impairments in parents of children with ASD are indicators of BAP (52). For example, children whose parents have subthreshold autistic traits show a significant shift toward more pathological scores in reciprocal social behavior. The continuous distribution of heritable subthreshold autism impairments is observed in both adults and children (53).

#### 4.2.3 Social functioning

In previous studies, AT are distributed continuously along a spectrum, ranging from non-clinical to clinical conditions (33). Although AT are less severe than those observed in individuals with autism in some cases (54–57), they are no denying closely associated with difficulties in social functioning, particularly in the formation and maintenance of interpersonal relationships, which can negatively impact an individual’s quality of life (58).

The research has concentrated on the behavioral and mental health consequences for individuals with AT. It is evident that AT can hinder fulfilling social interactions. Individuals with higher levels of AT are more prone to experiences of bullying, despair, anxiety, and loneliness (5, 59, 60). Wing (61) examined the relationship between AT and challenging behaviors, using a five-question framework that assessed lack of speech, social interaction, empathy, stereotypies, and routines. Although Wing’s study provided valuable insights into specific behavioral manifestations of AT, it did not address genetic etiology, brain function, or mental health. Chaplin et al. (62) found that individuals with elevated AT, compared to neurotypical prisoners, are more likely to report suicidal ideation, self-harm, and vulnerability to mental illnesses. Additional evidence links sleep problems and poor mental health with AT. For instance, Stewart et al. (63) reported that older adults with high AT experience less restful sleep and more mental health impairments than those with low AT. Similarly, Liisa Salmela et al. (64) found that individuals with high AT report shorter sleep durations at age of 16.

Research in AT primarily focuses on an individual’s social functioning, investigating the correlation between AT levels and factors such as sleep and depression. However, intervention-based studies in this area are limited, potentially due to the high costs involved. Future research should explore intervention strategies and measures to improve the psychological well-being and sleep quality of individuals with high AT levels, thereby enhancing the practical significance of AT, which is encouraged. Notably, in the realm of behavioral prediction, one study utilized effective transfer learning algorithms to predict autism spectrum disorder (ASD), suggesting that AI algorithms can be employed in research alongside traditional experimental methods (65).

#### 4.2.4 Geographical variations

We analyzed four countries (the UK, USA, Japan, and China) with significant contributions to autistic traits (AT) research. UK studies focus on gene-environment interactions, including twin studies, prenatal testosterone influences, and associations between autism and schizophrenia spectrum disorders (66, 67). Social cognition themes such as theory of mind, empathy deficits, and clinical vs. non-clinical group differences are also emphasized (68–70). US research spans broader environmental factors (e.g., prenatal pollution, chemical exposures), genetic explorations (e.g., SHANK3 mutations), neuroimaging techniques (fMRI, DTI), and comorbidity with conditions like ADHD and anxiety (71, 72). US studies also prioritize cross-cultural comparisons and large-scale epidemiological investigations, such as multinational cohort analyses (Fumiyo 73).

Japanese research frequently addresses sensory-perceptual processing, including cross-modal integration (visual-gustatory associations), temporal perception, and auditory processing (74, 75). Mental health topics, such as postpartum depression, occupational stress in pharmacists, and culturally specific phenomena like hikikomori (social withdrawal), are prominent (76). Additionally, Japanese studies focus on subclinical populations and non-clinical samples to examine AT manifestations in daily life (74, 77). Chinese investigations emphasize neurobiological mechanisms, such as gene variations (OXTR, IL-6), brain structural differences (gray/white matter connectivity), and oxytocin’s role (78–80). Social behaviors like camouflaging, emotional prosody recognition, and environmental factors (e.g., prenatal education, screen time) are also explored (81, 82). Gender differences in autism, particularly between females and males, constitute a key theme (83).

In methodological approaches, Chinese and Japanese studies predominantly employ behavioral experiments and localized assessment tools, prioritizing real-life interpersonal scenarios (84). In contrast, the UK and US utilize advanced neuroimaging and molecular techniques to investigate neural mechanisms and compile large-scale datasets (85, 86). East Asian research emphasizes AT’s impact on social functioning, advocating adaptive strategies like camouflaging (73), while Western studies focus on neurobiological foundations and therapeutic interventions (e.g., oxytocin administration, gene therapies) (87, 88). Collectively, these nations aim to elucidate genetic, neural, behavioral, and environmental mechanisms underlying AT and develop targeted intervention strategies.

## 5 Conclusion

This bibliometric review of the literature on AT over the past 20 years has provided a wealth of insight into the development of the field, global research hotspots, and progress. The analysis of 1,044 publications reveals a gradual increase in global research interest, with a significant expansion in the number of publications from just 1 in 1997 to 165 in 2023, highlighting the growing prominence of the field. The field’s growth accelerated markedly after 2019, with a notable increase from 57 in 2019 to 90 in 2020. The most rapid growth occurred between 2022 and 2023, when the number of publications increased from 120 to 165.

The primary research areas are psychology, education, social sciences, and health. The UK stands out as a major contributor, with extensive international, institutional, and individual collaborations driving the research forward. Initially focused on families with ASD, research now includes the general population, examining both suprathreshold and subthreshold autistic traits. Recent studies are exploring AT from genetic, brain function, and etiological perspectives, establishing a solid scientific foundation. The AQ remains a key tool in assessing AT, with ongoing efforts to refine its reliability and validity.

The existing literature on AT is extensive, reflecting its status as a current research hotspot. This prominence stems from the increasing specificity of research subjects and the breadth of research dimensions, encompassing cognitive and genetic aspects, each with numerous research questions. Current studies also demonstrate preliminary explorations of the boundaries of AT, such as its association with somatic symptom disorder (89). Investigating these relationships is a key focus in 2024, primarily at a theoretical level. A more extended research focus may build upon these theoretical foundations to develop practical and effective intervention strategies for specific populations, an area with limited existing literature.

This study offers a comprehensive and reliable overview of AT research, capturing its historical development and current trends. While it does not encompass all publications, it provides a representative sample that is invaluable for understanding the field’s challenges and opportunities. The findings highlight significant issues related to AQ, subthreshold autistic traits, and social functioning, and are likely to stimulate further in-depth research in these areas.

## References

[1] American Psychiatric Association. Diagnostic and Statistical Manual of Mental Disorders (5th ed). Arlington, VA: American Psychiatric Publishing (2013). doi: 10.1176/appi.books.9780890425596.

[2] Sucksmith E Roth I Hoekstra RA. Autistic traits below the clinical threshold: re-examining the broader autism phenotype in the 21st century. Neuropsychol Rev. (2011) 21:360–89. doi: 10.1007/s11065-011-9183-9 21989834

[3] Baron-Cohen S Wheelwright S Skinner R Martin J Clubley E. The autism-spectrum quotient (AQ): evidence from Asperger syndrome/high-functioning autism, males and females, scientists and mathematicians. J Autism Dev Disord. (2001) 31:5–17. doi: 10.1023/A:1005653411471 11439754

[4] Gökçen E Petrides KV Hudry K Frederickson N Smillie LD. Sub-threshold autism traits: The role of trait emotional intelligence and cognitive flexibility. Br J Psychol. (2014) 105:187–99. doi: 10.1111/bjop.12033 PMC4209115 24754807

[5] Kunihira Y Senju A Dairoku H Wakabayashi A Hasegawa T. ‘Autistic’ traits in non-autistic Japanese populations: relationships with personality traits and cognitive ability. J Autism Dev Disord. (2006) 36:553–66. doi: 10.1007/s10803-006-0094-1 16602034

[6] Takahashi J Tamaki K Yamawaki N. Autism spectrum, attachment styles, and social skills in university student. Creative Educ. (2013) 4:514–20. doi: 10.4236/ce.2013.48075

[7] Wenyun Z Shiwei Z Qianqian Z Yinglin G Weiwei P. Autistic traits influence pain empathy: The mediation role of pain-related negative emotion and cognition. Acta Psychol Sin. (2023) 55:1501–7. doi: 10.3724/SP.J.1041.2023.01501

[8] Bhaumik S Branford D Mcgrother C Thorp C. Autistic traits in adults with learning disabilities. Br J Psychiatry J Ment Sci. (1997) 170:502–6. doi: 10.1192/bjp.170.6.502 9330013

[9] Constantino J Todd R. Autistic traits in the general population - A twin study. Arch Gen Psychiatry. (2003) 60:524–30. doi: 10.1001/archpsyc.60.5.524 12742874

[10] Szatmari P Zwaigenbaum L Bryson S. Conducting genetic epidemiology studies of autism spectrum disorders: issues in matching. J Autism Dev Disord. (2004) 34:49–57. doi: 10.1023/b:jadd.0000018074.74369.cd 15098957

[11] Baron-Cohen S Hoekstra RA Knickmeyer R Wheelwright S. The autism-spectrum quotient (AQ)—Adolescent version. J Autism Dev Disord. (2006) 36:343–50. doi: 10.1007/s10803-006-0073-6 16552625

[12] Constantino JN Davis SA Todd RD Schindler MK Gross MM Brophy SL. Validation of a brief quantitative measure of autistic traits: comparison of the social responsiveness scale with the autism diagnostic interview-revised. J Autism Dev Disord. (2003) 33:427–33. doi: 10.1023/A:1025014929212 12959421

[13] Woodbury-Smith MR Robinson J Wheelwright S Baron-Cohen S. Screening adults for Asperger Syndrome using the AQ: a preliminary study of its diagnostic validity in clinical practice. J Autism Dev Disord. (2005) 35:331–5. doi: 10.1007/s10803-005-3300-7 16119474

[14] Happé F Ronald A. The ‘Fractionable autism triad’: A review of evidence from behavioural, genetic, cognitive and neural research. Neuropsychol Rev. (2008) 18:287–304. doi: 10.1007/s11065-008-9076-8 18956240

[15] Stewart ME Watson J Allcock AJ Yaqoob T. Autistic traits predict performance on the block design. Autism. (2009) 13:133–42. doi: 10.1177/1362361308098515 19261684

[16] Chakrabarti B Dudbridge F Kent L Wheelwright S Hill-Cawthorne G Allison C. Genes related to sex steroids, neural growth, and social-emotional behavior are associated with autistic traits, empathy, and Asperger syndrome. Autism Res. (2009) 2:157–77. doi: 10.1002/aur.80 19598235

[17] Edelson LR Saudino KJ. Genetic and environmental influences on autistic-like behaviors in 2-year-old twins. Behav Genet. (2009) 39:255–64. doi: 10.1007/s10519-009-9270-3 PMC4096860 19377871

[18] Yerys BE Wallace GL Sokoloff JL Shook DA James JD Kenworthy L. Attention deficit/hyperactivity disorder symptoms moderate cognition and behavior in children with autism spectrum disorders. Autism Res. (2009) 2:322–33. doi: 10.1002/aur.103 PMC3012375 19998356

[19] Carpita B Nardi B Pronestì C Parri F Giovannoni F Cremone IM. May female autism spectrum be masked by eating disorders, borderline personality disorder, or complex PTSD symptoms? A case series. Brain Sci. (2023) 14(1):37. doi: 10.3390/brainsci14010037 38248252 PMC10813290

[20] Tahıllıoğlu A Çelik D Huseynova S Satar A Ercan ES. The association between autistic-like traits and sluggish cognitive tempo symptoms in children with ADHD. Int J Dev Disabil. (2024) 70:1227–36. doi: 10.1080/20473869.2023.2170485 PMC11660407 39712435

[21] Denney AS Tewksbury R. How to write a literature review. J Crim Justice Educ. (2013) 24:218–34. doi: 10.1080/10511253.2012.730617

[22] Wiles R. What are qualitative research ethics? Bloomsbury Academic. (2012) 128. doi: 10.5040/9781849666558

[23] Singh VK Singh P Karmakar M Leta J Mayr P. The journal coverage of Web of Science, Scopus and Dimensions: A comparative analysis. Scientometrics. (2021) 126:5113–42. doi: 10.1007/s11192-021-03948-5

[24] Wang X Fang Z Sun X. Usage patterns of scholarly articles on Web of Science: a study on Web of Science usage count. Scientometrics. (2016) 109:917–26. doi: 10.1007/s11192-016-2093-0

[25] Wu MQ Wu DQ Hu CP Iao LS. Studies on children with developmental coordination disorder in the past 20 years: A bibliometric analysis. Front Psychiatry. (2021) 12:776883. doi: 10.3389/fpsyt.2021.776883 34938213 PMC8685384

[26] Yuan S Jing L Jiamiao H. Visual analysis of research progress of photodynamic sterilization technology based on citespace. Sci Technol Food Industry. (2024) 1–30. doi: 10.13386/j.issn1002-0306.2023010162

[27] Yan F Zhangkai W Xuewei Y Tianchu L Haiping G. A bibliometric analysis of antibiotic resistance genes in soil media based on web of science. Asian J Ecotoxicol. (2023) 18:302–13. doi: 10.7524/AJE.1673-5897.20230409001

[28] Chen C Hu Z Liu S Tseng H. Emerging trends in regenerative medicine: a scientometric analysis in CiteSpace. Expert Opin Biol Ther. (2012) 12:593–608. doi: 10.1517/14712598.2012.674507 22443895

[29] Zhou Q Kong HB He BM Zhou SY. Bibliometric analysis of bronchopulmonary dysplasia in extremely premature infants in the web of science database using CiteSpace software. Front Pediatr. (2021) 9:705033. doi: 10.3389/fped.2021.705033 34490163 PMC8417835

[30] Ruzich E Allison C Smith P Watson P Auyeung B Ring H. Measuring autistic traits in the general population: a systematic review of the Autism-Spectrum Quotient (AQ) in a nonclinical population sample of 6,900 typical adult males and females. Mol Autism. (2015) 6. doi: 10.1186/2040-2392-6-2 PMC4396128 25874074

[31] Bralten J van Hulzen KJ Martens MB Galesloot TE Arias Vasquez A Kiemeney LA. Autism spectrum disorders and autistic traits share genetics and biology. Mol Psychiatry. (2018) 23(5):1205–12. doi: 10.1038/mp.2017.98 PMC5984081 28507316

[32] Ma D Yang B Guan B Song L Liu Q Fan Y. A bibliometric analysis of Pyroptosis from 2001 to 2021. Front Immunol. (2021) 12:731933. doi: 10.3389/fimmu.2021.731933 34484243 PMC8416445

[33] Massullo C Imperatori C Adenzato M Brunetti R Ardito RB. Abnormal EEG power spectrum in individuals with high autistic personality traits: an eLORETA study. J Psychopathol Behav Assess. (2020) 42:560–9. doi: 10.1007/s10862-019-09777-4

[34] Carpita B Cremone I Amatori G Cappelli A Salerni A Massimetti G. Investigating the relationship between orthorexia nervosa and autistic traits in a university population. CNS Spectrums. (2022) 27:613–20. doi: 10.1017/S1092852921000420 33866990

[35] Dell’Osso L Cremone IM Carpita B Fagiolini A Massimetti G Bossini L. Correlates of autistic traits among patients with borderline personality disorder. Compr Psychiatry. (2018) 83:7–11. doi: 10.1016/j.comppsych.2018.01.002 29500962

[36] Dell’Osso L Amatori G Massimetti G Nardi B Gravina D Benedetti F. Investigating the relationship between autistic traits and symptoms and catatonia Spectrum. Eur Psychiatry. (2022) 65. doi: 10.1192/j.eurpsy.2022.2334 PMC9724219 36328964

[37] Dell’Osso L Cremone IM Chiarantini I Arone A Massimetti G Carmassi C. Autistic traits and camouflaging behaviors: a cross-sectional investigation in a University student population. CNS Spectrums. (2022) 27:740–6. doi: 10.1017/S1092852921000808 34505557

[38] Dell’Osso L Nardi B Benedetti F Cremone IM Casagrande D Massimetti G. Orthorexia and autism spectrum in University workers: relationship with gender, body mass index and dietary habits. Eating Weight Disord Stud Anorexia Bulimia Obes. (2022) 27:3713–23. doi: 10.1007/s40519-022-01514-3 36434469

[39] Lucia B Sara C Eugenia C Camilla G Claudia C Liliana DO. The broad autism (Endo)Phenotype: neurostructural and neurofunctional correlates in parents of individuals with autism spectrum disorders. Front Neurosci. (2016) 10:346. doi: 10.3389/fnins.2016.00346 27499732 PMC4956643

[40] Pini S Abelli M Carpita B Dell’Osso L Castellini G Carmassi C. Historical evolution of the concept of anorexia nervosa and relationships with orthorexia nervosa, autism, and obsessive-compulsive spectrum. Neuropsychiatr Dis Treat. (2016) 12:1651–60. doi: 10.2147/NDT.S108912 PMC4939998 27462158

[41] Fan L Springfield C Klein H Ackerman RA Sasson NJ Pinkham AE. Assessing the diametrical model of schizotypal and autistic traits in emotion recognition and social functioning in a community sample. Schizophr Res. (2023) 261:194–202. doi: 10.1016/j.schres.2023.09.038 37797360

[42] Wang J Lee LC Chen YS Hsu JW. Assessing autistic traits in a Taiwan preschool population: cross-cultural validation of the Social Responsiveness Scale (SRS). J Autism Dev Disord. (2012) 42:2450–9. doi: 10.1007/s10803-012-1499-7 22407579

[43] Skuse DH Mandy WP Scourfield J. Measuring autistic traits: heritability, reliability and validity of the Social and Communication Disorders Checklist. Br J Psychiatry. (2005) 187:568–72. doi: 10.1192/bjp.187.6.568 16319410

[44] Anyzova P Mateju P. Beauty still matters: The role of attractiveness in labour market outcomes. Int Sociol. (2018) 33(3):269–91. doi: 10.1177/0268580918760431

[45] Jian G Xudong Z. Sub-threshold autistic traits in normal population: its concept, structure and influencing factors. Adv psychol Sci. (2015) 9):1599. doi: 10.3724/SP.J.1042.2015.01599

[46] Carpita B Carmassi C Calderoni S Muti D Muscarella A Massimetti G. The broad autism phenotype in real-life: clinical and functional correlates of autism spectrum symptoms and rumination among parents of patients with autism spectrum disorder. CNS Spectr. (2020) 25(6):765–73. doi: 10.1017/S1092852919001615 31747980

[47] Carpita B Muti D Cremone I Fagiolini A Dell’Osso L. Eating disorders and autism spectrum: links and risks. CNS Spectrums. (2020) 27:272–80. doi: 10.1017/S1092852920002011 33161925

[48] Bailey A Palferman S Heavey L Couteur AL. Autism: the phenotype in relatives. J Autism Dev Disord. (1998) 28:369–92. doi: 10.1023/A:1026048320785 9813774

[49] Constantino JN Lajonchere C Lutz M Gray T Todd RD. Autistic social impairment in the siblings of children with pervasive developmental disorders. Am J Psychiatry. (2006) 163:294–6. doi: 10.1176/appi.ajp.163.2.294 16449484

[50] Xiao X Na Y Le-Qiong Q Shi-Jie Z. Personality, empathy and broad autism phenotype of parents of the children with autism. Chin J Clin Psychol. (2014) 22. doi: 10.16128/j.cnki.1005-3611.2014.01.012

[51] Wheelwright S Auyeung B Allison C Baron-Cohen S. Defining the broader, medium and narrow autism phenotype among parents using the Autism Spectrum Quotient (AQ). Mol Autism. (2010) 1:10. doi: 10.1186/2040-2392-1-10 20678260 PMC2913943

[52] Kose S Bora E Erermiş S Özbaran B Bildik T Aydın C. Broader autistic phenotype in parents of children with autism: Autism Spectrum Quotient-Turkish version. Psychiatry Clin Neurosci. (2013) 67:20–7. doi: 10.1111/pcn.12005 23331285

[53] Constantino JN Todd RD. Intergenerational transmission of subthreshold autistic traits in the general population. Biol Psychiatry. (2005) 57:655–60. doi: 10.1016/j.biopsych.2004.12.014 15780853

[54] August GJ Stewart MA Tsai L. The incidence of cognitive disabilities in the siblings of autistic children. Br J Psychiatry. (1981) 138:416–22. doi: 10.1192/bjp.138.5.416 6456787

[55] Parr JR Wittemeyer K Le Couteur AS. Commentary: the broader autism phenotype implications for research & Clinical practice. In: Amaral D Geschwind D Dawson G eds. Autism Spectrum Disorders. (2011) (New York: Oxford Academic), 521–4. doi: 10.1093/med/9780195371826.003.0034

[56] Piven J Wzorek M Landa R Lainhart J Folstein SE. Personality characteristics of the parents of autistic individuals. psychol Med. (1994) 24:783–95. doi: 10.1017/S0033291700027938 7991760

[57] Wolff S Narayan S B. M. Personality characteristics of parents of autistic children. J Child Psychol Psychiatry. (1988) 29:143–53. doi: 10.1111/j.1469-7610.1988.tb00699.x 3372611

[58] Pisula E Danielewicz D Kawa R Pisula W. Autism spectrum quotient, coping with stress and quality of life in a non-clinical sample – an exploratory report. Health Qual Life Outcomes. (2015) 13:173. doi: 10.1186/s12955-015-0370-x 26503411 PMC4624179

[59] Jobe LE White SW. Loneliness, social relationships, and a broader autism phenotype in college students. Pers Individ Dif. (2007) 42:1479–89. doi: 10.1016/j.paid.2006.10.021

[60] Lamport D Zlomke KR. The broader autism phenotype, social interaction anxiety, and loneliness: implications for social functioning. Curr Psychol. (2014) 33:246–55. doi: 10.1007/s12144-014-9210-0

[61] Wing L. (1988). Aspects of Autism: Biological Research : Proceedings of a Conference Held at the University of Kent, 18-20 September, 1987. (Royal College of Psychiatrists).

[62] Chaplin E Mccarthy J Allely CS Forrester A Murphy D. Self-harm and Mental Health Characteristics of Prisoners with elevated rates of autistic traits. Res Dev Disabil. (2021) 114:103987. doi: 10.1016/j.ridd.2021.103987 34004498

[63] Stewart GR Corbett A Ballard C Creese B Aarsland D Hampshire A. Sleep problems and mental health difficulties in older adults who endorse high autistic traits. Res Autism Spectr Disord. (2020) 77:101633. doi: 10.1016/j.rasd.2020.101633

[64] Salmela L Kuula L Merikanto I Räikkönen K Pesonen AK. Autistic traits and sleep in typically developing adolescents. Sleep Med. (2019) 54:164–71. doi: 10.1016/j.sleep.2018.09.028 30580189

[65] Vasant Bidwe R Mishra S Kamini Bajaj S Kotecha K. Attention-focused eye gaze analysis to predict autistic traits using transfer learning. Int J Comput Intell Syst. (2024) 17:120. doi: 10.1007/s44196-024-00491-y

[66] Dooley N Ruigrok A Holt R Allison C Tsompanidis A Waldman J. Is there an association between prenatal testosterone and autistic traits in adolescents? Psychoneuroendocrinology. (2022) 136:105623. doi: 10.1016/j.psyneuen.2021.105623 34896742 PMC8783053

[67] Georgiou N Spanoudis G. Developmental language disorder and autism: commonalities and differences on language. Brain Sci. (2021) 11(5):589. doi: 10.3390/brainsci11050589 33946615 PMC8147217

[68] Coombs E Brosnan M Bryant-Waugh R Skevington SM. An investigation into the relationship between eating disorder psychopathology and autistic symptomatology in a non-clinical sample. Br J Clin Psychol. (2011) 50:326–38. doi: 10.1348/014466510x524408 21810110

[69] Hambrook D Tchanturia K Schmidt U Russell T Treasure J. Empathy, systemizing, and autistic traits in anorexia nervosa: a pilot study. Br J Clin Psychol. (2008) 47:335–9. doi: 10.1348/014466507x272475 18208640

[70] Kung KTF. Autistic traits, systemising, empathising, and theory of mind in transgender and non-binary adults. Mol Autism. (2020) 11:73. doi: 10.1186/s13229-020-00378-7 32993801 PMC7523342

[71] Guxens M Ghassabian A Gong T Garcia-Esteban R Porta D Giorgis-Allemand L. Air pollution exposure during pregnancy and childhood autistic traits in four European population-based cohort studies: the ESCAPE project. Environ Health Perspect. (2016) 124:133–40. doi: 10.1289/ehp.1408483 PMC4710593 26068947

[72] Oulhote Y Lanphear B Braun JM Webster GM Arbuckle TE Etzel T. Gestational exposures to phthalates and folic acid, and autistic traits in Canadian children. Environ Health Perspect. (2020) 128:27004. doi: 10.1289/ehp5621 32073305 PMC7064316

[73] Oshima F Takahashi T Tamura M Guan S Seto M Hull L. The association between social camouflage and mental health among autistic people in Japan and the UK: a cross-cultural study. Mol Autism. (2024) 15:1. doi: 10.1186/s13229-023-00579-w 38178255 PMC10768303

[74] Kondo HM Lin IF. Excitation-inhibition balance and auditory multistable perception are correlated with autistic traits and schizotypy in a non-clinical population. Sci Rep. (2020) 10:8171. doi: 10.1038/s41598-020-65126-6 32424307 PMC7234986

[75] Tsuji Y Imaizumi S. Autistic traits and speech perception in social and non-social noises. Sci Rep. (2024) 14:1414. doi: 10.1038/s41598-024-52050-2 38228768 PMC10791598

[76] Shimono Y Hasegawa A Tsuchihara K Tanaka K Matsuda Y Kunisato Y. Longitudinal association between autistic traits and affinity for hikikomori in Japanese university students. Curr Psychol. (2022) 41:8842–9. doi: 10.1007/s12144-020-01287-x

[77] Liu X Kawamura Y Shimada T Otowa T Koishi S Sugiyama T. Association of the oxytocin receptor (OXTR) gene polymorphisms with autism spectrum disorder (ASD) in the Japanese population. J Hum Genet. (2010) 55:137–41. doi: 10.1038/jhg.2009.140 20094064

[78] Montag C Sindermann C Melchers M Jung S Luo R Becker B. A functional polymorphism of the OXTR gene is associated with autistic traits in Caucasian and Asian populations. Am J Med Genet B Neuropsychiatr Genet. (2017) 174:808–16. doi: 10.1002/ajmg.b.32596 29027364

[79] Xu XJ Shou XJ Li J Jia MX Zhang JS Guo Y. Mothers of autistic children: lower plasma levels of oxytocin and Arg-vasopressin and a higher level of testosterone. PloS One. (2013) 8:e74849. doi: 10.1371/journal.pone.0074849 24086383 PMC3783493

[80] Yaxu Y Ren Z Ward J Jiang Q. Atypical brain structures as a function of gray matter volume (GMV) and gray matter density (GMD) in young adults relating to autism spectrum traits. Front Psychol. (2020) 11:523. doi: 10.3389/fpsyg.2020.00523 32322224 PMC7158890

[81] Chen J Strodl E Huang LH Chen JY Liu XC Yang JH. Associations between prenatal education, breastfeeding and autistic-like behaviors in pre-schoolers. Children (Basel). (2021) 8(2):124. doi: 10.3390/children8020124 33572414 PMC7916179

[82] Chen JY Strodl E Wu CA Huang LH Yin XN Wen GM. Screen time and autistic-like behaviors among preschool children in China. Psychol Health Med. (2021) 26:607–20. doi: 10.1080/13548506.2020.1851034 33227216

[83] Dai M Lin L Liang J Wang Z Jing J. Gender difference in the association between executive function and autistic traits in typically developing children. J Autism Dev Disord. (2019) 49:1182–92. doi: 10.1007/s10803-018-3813-5 30443698

[84] Stickley A Tachibana Y Hashimoto K Haraguchi H Miyake A Morokuma S. Assessment of autistic traits in children aged 2 to 4½ Years with the preschool version of the social responsiveness scale (SRS-P): findings from Japan. Autism Res. (2017) 10:852–65. doi: 10.1002/aur.1742 PMC6586029 28256099

[85] Rubinstein M Han S Tai C Westenbroek RE Hunker AC Scheuer T. Dissecting the phenotypes of Dravet syndrome by gene deletion. Brain: A J Neurol. (2015) 138 Pt 8:2219–33. doi: 10.1093/brain/awv142 PMC5022661 26017580

[86] Sano M Hirosawa T Yoshimura Y Hasegawa C An KM Tanaka S. Neural responses to syllable-induced P1m and social impairment in children with autism spectrum disorder and typically developing Peers. PloS One. (2024) 19:e0298020. doi: 10.1371/journal.pone.0298020 38457397 PMC10923473

[87] Ronald A Butcher LM Docherty S Davis OSP Schalkwyk LC Craig IW. A genome-wide association study of social and non-social autistic-like traits in the general population using pooled DNA, 500 K SNP microarrays and both community and diagnosed autism replication samples. Behav Genet. (2010) 40:31–45. doi: 10.1007/s10519-009-9308-6 20012890 PMC2797846

[88] Ronald A HappÉ F Price TS Baron-Cohen S Plomin R. Phenotypic and genetic overlap between autistic traits at the extremes of the general population. J Am Acad Child Adolesc Psychiatry. (2006) 45:1206–14. doi: 10.1097/01.chi.0000230165.54117.41 17003666

[89] Carpita B Nardi B Tognini V Poli F Amatori G Cremone IM. Autistic traits and somatic symptom disorders: what is the link? Brain Sci. (2024) 14(3):274. doi: 10.3390/brainsci14030274 38539662 PMC10968945

